# Resin Infiltration of Non-Cavitated Proximal Caries Lesions in Primary and Permanent Teeth: A Systematic Review and Scenario Analysis of Randomized Controlled Trials

**DOI:** 10.3390/jcm12020727

**Published:** 2023-01-16

**Authors:** Marcus Cebula, Gerd Göstemeyer, Joachim Krois, Vinay Pitchika, Sebastian Paris, Falk Schwendicke, Susanne Effenberger

**Affiliations:** 1 Clinical Research Department, DMG Dental Material Gesellschaft mbH, Elbgaustraße 248, 22547 Hamburg, Germany; 2 Department of Restorative, Preventive and Pediatric Dentistry, Charité—Universitätsmedizin Berlin, Aßmannshauser Str. 4-6, 14197 Berlin, Germany; 3 Department of Oral Diagnostics, Digital Health and Health Services Research, Charité—Universitätsmedizin Berlin, Aßmannshauser Str. 4-6, 14197 Berlin, Germany

**Keywords:** caries, biomaterials, resin infiltration, systematic reviews, evidence-based dentistry, clinical studies/trials

## Abstract

The present study aimed to meta-analyze and evaluate the certainty of evidence for resin infiltration of proximal carious lesions in primary and permanent teeth. While resin infiltration has been shown efficacious for caries management, the certainty of evidence remains unclear. The protocol was registered with PROSPERO (CRD42018080895), and PRISMA guidelines have been followed. The databases PubMed, Embase, and Cochrane CENTRAL were systematically screened, complemented by hand searches and cross-referencing. Eleven relevant articles were identified and included, i.e., randomized controlled trials (RCTs) comparing the progression of resin infiltrated proximal caries lesions (combined with non-invasive measures) in primary or permanent teeth with non-invasive measures. Random-effects meta-analyses and trial sequential analyses (TSA) were performed for per-protocol (PP), intention-to-treat (ITT), and best/worst case (BC/WC) scenarios. Six included trials assessed lesions in permanent teeth and five trails assessed lesions in primary teeth. The trials had a high or unclear risk of bias. Risk of caries progression was significantly reduced for infiltrated lesions in the PP, ITT, and BC scenarios in both permanent teeth and primary teeth, but not in the WC scenario. According to the TSA, firm evidence was reached for all of the scenarios except the WC. In conclusion, there is firm evidence for resin infiltration arresting proximal caries lesions in permanent and primary teeth.

## 1. Introduction

While a considerable reduction in the prevalence of cavitated caries lesions has been observed in many countries worldwide [1,2,3,4], dental caries remains one of the most prevalent diseases of humankind, affecting elderly and young people alike [5]. Caries is a dynamic and continuous process that, if uncontrolled, eventually leads to cavitation and the need for restorative treatment to maintain the form and function of the tooth. Initiating restoration, however, initiates an escalating cycle of increasingly invasive re-treatments (restorative spiral), which is why contemporary caries management focusses on preventing, arresting, and possibly reversing the caries process to delay or completely avoid restorative treatment [6,7,8,9,10].

Proximal surfaces are particularly susceptible to caries [11,12,13], with restorations of proximal lesions also requiring more invasive treatment while showing lower survival rates compared to restorations of non-proximal lesions. Hence, early interventions, i.e., non-invasive and micro-invasive strategies, aiming to prevent restorative treatments for these surfaces are particularly relevant to maintain primary and permanent teeth [9,14].

Non-invasive strategies include re-mineralization treatments (for example via topical fluoridation) as well as oral hygiene improvement measures, i.e., dietary advice and biofilm control. While they are simple to implement and preserve dental tissue, poor patient compliance may compromise their effectiveness [7,10,15]. An alternative is micro-invasive strategies, which are less reliant on patients’ compliance. These strategies aim to erect diffusion barriers either within (resin infiltration) or onto (sealing) the demineralized tissue to block acid diffusion, thereby inhibiting caries lesion progression. The need to remove a few micrometers of tissue for conditioning renders these treatments “micro-invasive”, while they are still more conservative compared to regular invasive treatment [10,16].

Over the last few years, multiple systematic reviews and meta-analyses have highlighted the efficacy of micro-invasive treatments compared to non-invasive treatments. In particular, resin infiltration has been recommended for arresting proximal caries lesions [10,16,17,18,19,20,21,22,23]. However, the certainty of this evidence, including the influence of data missing due to attrition, remains unclear. Furthermore, it is not clear how different dentitions influence the efficacy of resin infiltration. Considering the structural differences between permanent and primary teeth, with the latter having a higher potential for caries and caries progression, evaluating the efficacy of resin infiltration in both dentitions separately may give valuable information on the robustness of the evidence and derived recommendations.

The objective of this systematic review was to assess the evidence of the most recent follow-up reports of clinical trials on the efficacy of resin infiltration (in combination with non-invasive methods) in treating proximal caries compared to non-invasive strategies alone. Meta-analyses were conducted based on dentition type and missing data scenarios, i.e., per-protocol (PP), intention-to-treat (ITT), and best-case/worst-case (BC/WC, see below), to assess the strength and robustness of the data and to evaluate the uncertainty introduced by missing data. Moreover, trial sequential analysis (TSA) was used to evaluate whether the conclusions are based on quantitatively sufficient data.

## 2. Materials and Methods

This review is based on a protocol registered in the PROSPERO database (CRD42018080895) and the recommendations of the PRISMA statement for the reporting of systematic reviews have been followed [24]. We deviated from the protocol considering the assessed groups, not including studies on sealing and only focusing on resin infiltration.

### 2.1. Search Strategy

**Figure 1:**
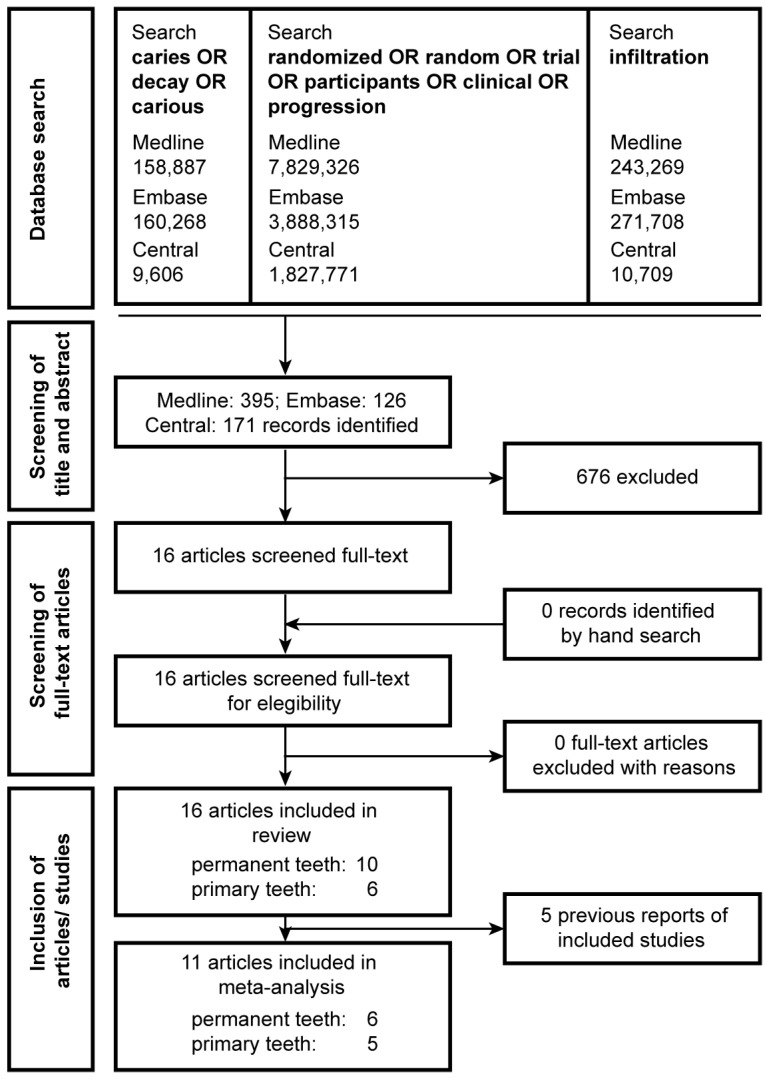
PRISMA flowchart of study selection. Search terms are indicated in the upper boxes, including the number of studies yielded for the three search groups. A combination of the three groups resulted in the number of records shown below the upper boxes. Five reports were excluded from the meta-analysis as they were earlier follow-ups of the included studies.

To identify potentially relevant studies, three electronic databases (Embase, Medline, and Cochrane Central) were searched independently by two of the authors (GG and SE) using a defined search protocol (as outlined in Figure 1). The search was conducted in November 2021. No filters were applied in the searches and language, and the year of publication was not restricted. Additionally, a complementary hand search was performed and references from selected publications were screened to search for relevant articles that did not appear in the database search. Neither the authors nor the journals were blinded to reviewers.

### 2.2. Inclusion Criteria

The search strategy and inclusion criteria were based on the PICOS framework:

- Population (P): Children and adults with non-cavitated proximal carious lesions (presumed intact surface status) in either permanent or primary teeth.
- Intervention (I): Infiltration of proximal caries.
- Comparison (C): Non-invasive strategies (e.g., oral hygiene and dietary control, topical fluoride, etc.) and/or placebo treatment should have been used for comparison to the intervention (infiltration).
- Outcome (O): Caries lesion progression detected by radiographs (digital subtraction radiography (DSR) or, if not available, pairwise reading or, if not available, lesion staging (for example according to radiographic enamel depth).
- Study design (S): Randomized controlled trials (RCTs) with either a parallel group or a split-mouth design.

### 2.3. Study Selection

The titles and the abstracts of the identified records were screened by two of the authors (GG and SE) based on the PICOS framework. In addition, only articles in English and available in full text were considered. If either author found a record to be potentially eligible, the full text was assessed independently by both of the authors with inclusion having been decided again by the two reviewers based on the inclusion criteria (PICOS criteria). Consensus was obtained by discussion, or in consultation with a third author (MC). For studies with multiple reports, only the last one with the longest follow-up period was considered for the meta-analysis.

### 2.4. Data Extraction and Risk of Bias

Data extraction was performed independently by two of the authors (GG and SE) and the following items were extracted and recorded: the study design, publication year, setting (e.g., dental school or practice); source of funding and declaration of interests; sample (age, number of patients, dentition, baseline caries experience, or caries risk); interventions (infiltration and control treatment description, including the number of teeth treated); and outcomes (method of assessment, drop-outs, follow-up, and failures). Only the latest reported follow-up time point was used for analysis. The authors of original studies were contacted for data clarification if needed. The risk of bias was assessed by two independent reviewers (GG and SE) as outlined in the Cochrane handbook for systematic reviews of interventions [25], with a separate assessment of blinding of operators/participants and examiners. Disagreements were resolved by discussion or in consultation with a third author (MC).

### 2.5. Effective Sample Size

All of the included studies used a split-mouth design. As some studies evaluated multiple lesion-pairs within a participant, we considered the individual person to be the statistical unit, whereas the lesions within an individual were viewed as clustered. Clustering effects were accounted for by reducing the size of each study to its effective sample size using the design effect [26] as described previously [23,27]. Essentially, the design effect is calculated as follows: 1 + (M − 1) × ICC, with M being the average cluster size (for split-mouth: the number of lesion pairs divided by the number of patients) and ICC referring to the intra-cluster correlation coefficient (set to 0.2 according to Masood et al. [28]). The effective sample size is then computed by dividing the number of lesions and events by the design effect.

### 2.6. Data Synthesis and Statistical Heterogeneity

Pairwise meta-analyses were performed via random-effects models using the metafor package [29] in R (R Foundation for Statistical Computing, 2017) as described previously [23]. Considering that all of the included studies employed a split-mouth design and reported binary outcomes, the Becker–Balagtas method [30,31] (setting ICC to 0.2 [28]) was applied to compute odds ratios (ORs) and 95% confidence intervals (95% CI), which were illustrated using Forest plots.

Meta-analyses were performed for the following scenarios:

- Intention-to-treat (ITT): All of the participants who received initial treatment regardless of their availability at follow-up are included, which is considered the most appropriate and least biased data analysis approach [26,32,33].
- Per-protocol (PP): Only participants who completed the treatment originally allocated and were followed up are included. This scenario aims to account for possible bias introduced by attrition and protocol deviations [34].
- Best-case (BC) and worst-case (WC) scenarios: In the best-case scenario all of the missing participants were assumed to have a favorable outcome in the resin infiltration group and a poor outcome in the control group, whereas the worst-case scenario assumes the converse. This approach is an imputation technique aiming to validate data reliability and robustness by providing an interval that includes all of the uncertainty related to the missing data [35,36].

Heterogeneity was assessed using the Chi^2^ test, with *p* < 0.1 being considered indicative of significant heterogeneity [37]. Additionally, I^2^-statistics were undertaken to quantify the extent of heterogeneity [38]. Publication bias was also assessed through funnel plots and Egger’s regression test for asymmetry using the regtest function in the metafor package using R [39]. Because there were few studies per analysis, the alpha level was set at 0.10. Therefore, a *p*-value of <0.10 was considered statistically significant, thereby indicating the presence of publication bias.

### 2.7. Quality of the Evidence

The evidence was graded according to the Grading of Recommendations, Assessment, Development, and Evaluation (GRADE) working group of evidence using GRADEpro (online software) [40,41,42]. According to GRADE, evidence is rated as high, moderate, low, and very low. High: further research is very unlikely to change our confidence in the estimated effect; moderate: further research is likely to have an important impact on our confidence in the estimated effect and may change the estimate; low: further research is very likely to have an important impact on our confidence in the estimated effect and is likely to change the estimate; and very low: any estimated effect is very uncertain. Assessment is made in relation to the risk of bias, inconsistency, indirectness, imprecision, and publication bias. To assess imprecision, the results of the TSA analysis were taken into consideration, i.e., whether the required information size was reached or not. [43]

### 2.8. Trial Sequential Analysis (TSA)

Meta-analyses may lead to false conclusions, i.e., by including too few participants [44] or through repeated significance testing in updated meta-analyses which is known to inflate the type I error leading to false-positive findings [45]. To control for such errors and to evaluate the actual amount of evidence that has been accrued, TSA was conducted. Essentially, trials are hereby included in chronological order and cumulated Z-values, i.e., test statistic, used to compare two interventions, with Z = 0 indicating no difference and Z exceeding ±1.96 indicating statistically significant difference (*p* ≤ 0.05, two-sided test). Z-values are calculated and plotted against the accumulated number of patients [46,47]. The resulting Z-curve is assessed regarding its relation to the conventional significance boundaries (Z = ±1.96), the required information size (RIS), and the trial sequential monitoring boundaries (TSMB), including superior/inferior as well as futility (non-superior/non-inferior) boundaries (FB).

Risk of type I error was set at α = 0.05. Risk of type II error was set at β = 0.20 equivalent to a power of 0.80. For calculation of the required IS and TSMB, the incidence rate in the control arm was calculated for each scenario individually, whereas a relative risk reduction (RRR) of 30% was assumed for all scenarios as a priori defined worthwhile interventional effect [46,48]. Furthermore, RIS was adjusted for heterogeneity/diversity (diversity-adjusted RIS: DARIS) using a DerSimonian–Laird estimator. The Lan–DeMets version [49] of the O’Brien–Fleming function [50] was used for determining TSMB.

Results crossing the conventional boundary of significance (Z = ±1.96) but not the superiority/inferiority TSMB were defined as spuriously significant. Firm evidence of superiority or inferiority was assumed when the Z-curve crossed the superiority or inferiority TSMB before DARIS was reached. Firm evidence of futility was confirmed by the Z-curve crossing the futility TSBM. TSA 0.9.5.10 Beta (Copenhagen Trial Unit, Copenhagen, Denmark) was used [51], and analyses were again performed for intention-to-treat and per-protocol as well as best-case and worst-case scenarios.

## 3. Results

### 3.1. Search Details

Of 692 records identified via database screening and cross-referencing, 16 were evaluated in full text. Five records were previous reports of included studies [52,53,54,55,56] and were thus excluded from the meta-analysis, leading to the inclusion of 11 reports for qualitative and quantitative synthesis (Figure 1, Table S1).

### 3.2. Study Characteristics

The characteristics of the included studies are summarized in Table S1. All of the studies were randomized controlled trials with a split-mouth design and originated from various countries: Germany (2), Turkey (1), the USA (2), Brazil (3), Columbia (1), New Zealand (1), and Greenland (1). The majority (8 out of 11 studies) were conducted in dental school settings (university clinics). Two studies were performed in public health or community clinics, and one was a practice-based trial. The reports on these studies were published between 2010 and 2021.

Of the eleven included studies, six assessed lesions in permanent teeth (270 patients, 802 lesions) [57,58,59,60,61,62] with follow-ups ranging from 12 to 84 months (median: 36 months) and yearly drop-outs of 1–15% (median: 3%). The participants in these studies were young adults (mean age of 20 to 26), with a predominantly mixed caries risk. Only one study included only high-risk patients and one did not state the caries risk at all.

The remaining five studies assessed lesions in primary teeth (261 patients, 522 lesions) [63,64,65,66,67]. Follow-up ranged between 12 to 24 months (median: 24 months) with a yearly drop-out of 2–11% (median: 10%). The studies included mixed caries risk children (mean age between 6 to 8).

The primary outcome in all of the studies was assessed using radiographic lesion progression. If more than one method was used for evaluation, only the most sensitive outcome was included in this meta-analysis, i.e., DSR or, if not available, pairwise reading of radiographs or, if not available, lesion staging (i.e., due to independent reading of radiographs) [16,68].

### 3.3. Risk of Bias

The risk of bias in all of the included studies is summarized in Table S2. Sequence generation was well reported in all of the studies, while the method of allocation concealment was not indicated in four studies [61,62,63,64]. In addition, one study did not indicate who performed the randomization [58]. None of the included studies clearly reported blinding of the personnel, which may have had an impact. However, considering that all of the studies were of a split-mouth design and that the concomitant treatments (i.e., oral hygiene and dietary instruction or topical fluoride) are essentially caries preventive measures addressing all teeth and the oral health of the patients in general, substantial treatment bias is rather unlikely. With regards to blinding of the participants, it has to be noted that five out of the six studies that assessed lesions in permanent teeth used sham treatment to blind participants [57,59,60,61,62], whereas only one study refrained from doing so [58]. In contrast, studies that included children (i.e., assessing lesions in primary teeth) generally refrained from using sham treatments, which may had had an impact on the outcome. Only one study assessing lesions in primary teeth reported the blinding of participants through keeping parents some distance away, while also assuming that the children were likely unaware which tooth was treated due to their age [67]. Blinding of the outcome assessment was performed and adequately described in all of the studies. Attrition bias was not suspected in most studies. However, three studies assessing lesions in permanent teeth failed to report the reason for the drop-outs [58,60,61] and two studies assessing lesions in primary teeth reported a rather high drop-out rate (over 40% after 24 months) [64,65] which is explained by exfoliation, but this may affect the study result. Bias due to reporting was rated low in four studies [58,62,64,66] and unclear in seven studies [57,59,60,61,63,65,67]. Of these, one study [60] reported pairwise reading as the primary endpoint in contrast to the pre-specified outcome digital subtraction radiography, whereas no study protocol was available for the other six studies. The majority of the studies (7 out of 11) were either sponsored or financially supported by the manufacturer [57,59,60,62,64,66,67]. Of these, three studies were suspected of industry bias as they were either conducted by the inventors of the product [57,59] or as sponsor funding appeared to have influenced the presentation of the results [62]. Furthermore, two studies were rated as having an unclear risk of other bias. One study failed to report the number of treated lesions [58], whereas the other study presented an unbalanced allocation of lesion severity [67], which was to the expense of the treatment group, however. Due to the small number of studies included in this review, meta-analyses were performed by pooling all of the studies together and they were not stratified based on the risk of bias assessment scores.

### 3.4. Meta-Analysis and TSA

**Figure 2:**
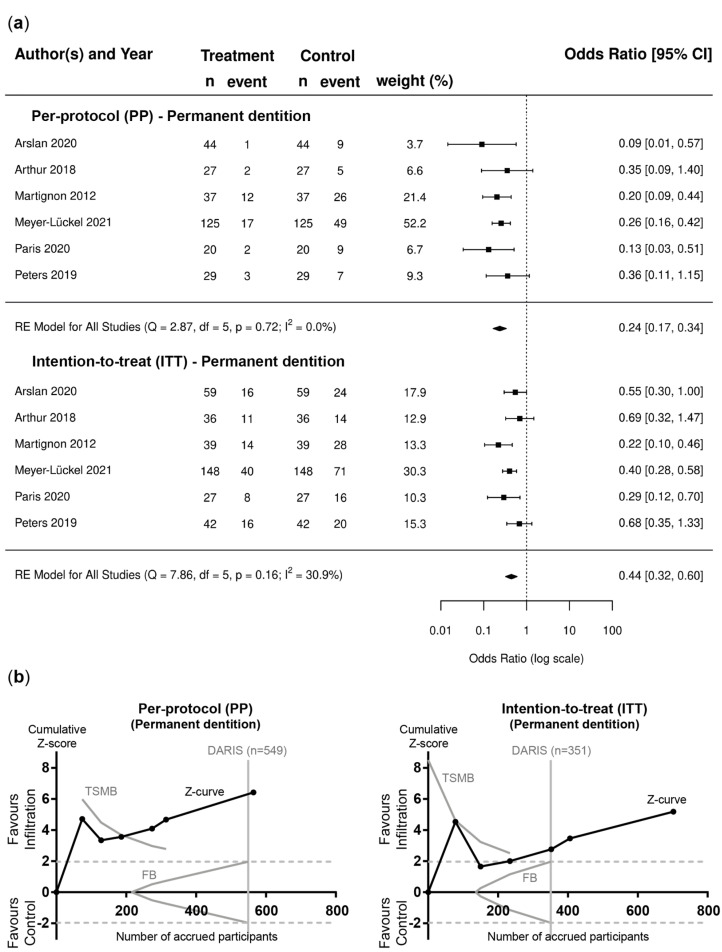
Permanent dentition. (**a**) Forest plots of pairwise meta-analysis for PP and ITT scenarios. Risk of failure, i.e., lesion progression, is indicated for lesions in permanent teeth treated with resin infiltration and non-invasive measures compared to placebo and non-invasive measures [57,58,59,60,61,62]. The odds ratio (OR) and 95% confidence intervals (CI) are shown. Heterogeneity is indicated by I^2^ and Q statistics. Diamonds indicate pooled effect estimates. The number of lesions was adjusted using the design effect as described. (**b**) Trial sequential analysis (TSA) of trials evaluating lesions in permanent teeth [57,58,59,60,61,62] for PP and ITT scenarios to assess the robustness of evidence. The cumulative Z-score (black curve), i.e., the accumulated level of significance, was plotted against the number of participants accrued so far, which was compared with the heterogeneity-adjusted required information size (DARIS), the conventional border (Z = 1.96), the trial sequential monitoring boundary (TSMB), and the futility border (FB) (grey oblique). For both scenarios, i.e., PP and ITT, the Z-curve crossed the conventional border and TSMB before reaching DARIS.

**Figure 3:**
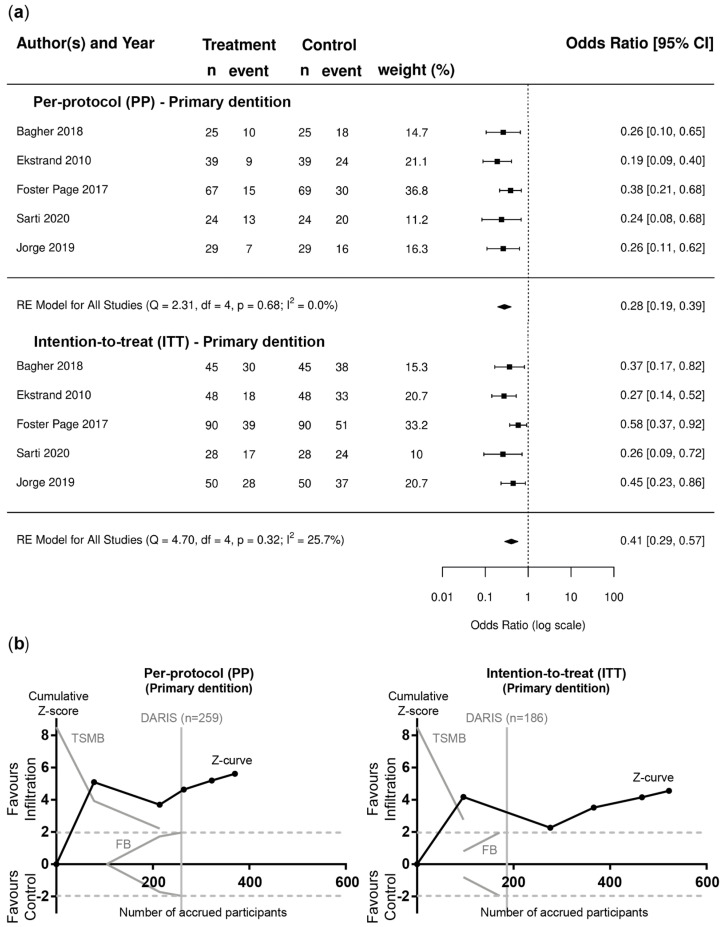
Primary dentition. (**a**) Forest plots of pairwise meta-analysis for PP and ITT scenarios. Risk of failure, i.e., lesion progression, is indicated for lesions in primary teeth treated with resin infiltration and non-invasive measures compared to non-invasive measures alone [63,64,65,66,67]. The odds ratio (OR) and 95% confidence intervals (CI) are shown. Heterogeneity is indicated by I^2^ and Q statistics. Diamonds indicate pooled effect estimates. The number of lesions was adjusted using the design effect as described. (**b**) Trial sequential analysis (TSA) of trials evaluating lesions in primary teeth [63,64,65,66,67] for PP and ITT scenarios to assess the robustness of evidence. The cumulative Z-score (black curve), i.e., the accumulated level of significance, was plotted against the number of participants accrued so far, which was compared with the heterogeneity-adjusted required information size (DARIS), the conventional border (Z = 1.96), the trial sequential monitoring boundary (TSMB), and the futility border (FB) (grey oblique). For both scenarios, i.e., PP and ITT, the Z-curve crossed the conventional border and TSMB before reaching DARIS.

Based on pairwise meta-analyses, the superiority of resin infiltration combined with non-invasive measures over non-invasive measures alone was found in PP and ITT analysis for lesions in permanent teeth (OR [95%CI]; PP: 0.24 [0.17–0.34]; ITT: 0.44 [0.32–0.60]) (Figure 2a) and primary teeth (OR [95%CI]; PP: 0.28 [0.19–0.39]; ITT: 0.41 [0.29–0.57]) (Figure 3a). TSA confirmed that the required information size was reached, with Z-curves crossing the conventional border and TSMB before reaching DARIS (Figure 2b and Figure 3b). Funnel plots and Egger’s regression test for asymmetry did not indicate publication bias (Figure S1) and no evidence of statistically significant heterogeneity was found for these comparisons (PP: I^2^ = 0%; Figure 2a and Figure 3a; ITT: I^2^ = 30.9% (permanent dentition) and I^2^ = 25.7% (permanent dentition)).

**Figure 4:**
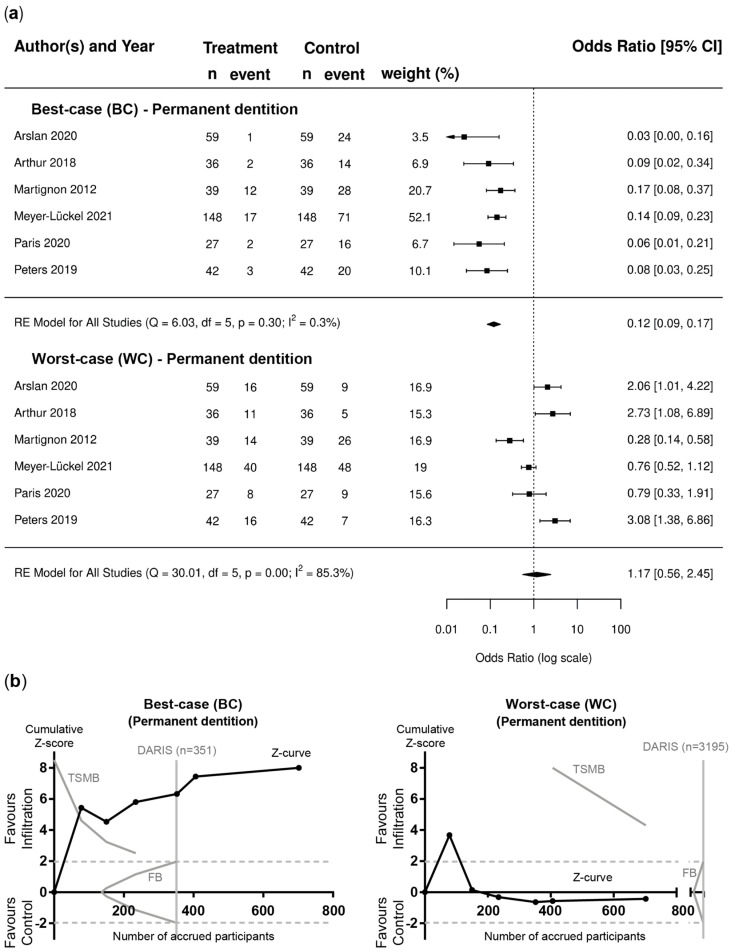
Permanent dentition. (**a**) Forest plots of pairwise meta-analysis for BC and WC scenarios. Risk of failure, i.e., lesion progression, is indicated for lesions in permanent teeth treated with resin infiltration and non-invasive measures compared to placebo and non-invasive measures [57,58,59,60,61,62]. The odds ratio (OR) and 95% confidence intervals (CI) are shown. Heterogeneity is indicated by I^2^ and Q statistics. Diamonds indicate pooled effect estimates. The number of lesions was adjusted using the design effect as described. (**b**) Trial sequential analysis (TSA) of trials evaluating lesions in permanent teeth [57,58,59,60,61,62] for BC and WC scenarios to assess the robustness of evidence. The cumulative Z-score (black curve), i.e., the accumulated level of significance, was plotted against the number of participants accrued so far, which was compared with the heterogeneity-adjusted required information size (DARIS), the conventional border (Z = 1.96), the trial sequential monitoring boundary (TSMB), and the futility border (FB) (grey oblique). For the BC scenarios the Z-curve crossed the conventional border and TSMB before reaching DARIS, whereas for the WC scenario, the Z-curve failed to cross the conventional border, TSMB, and DARIS.

**Figure 5:**
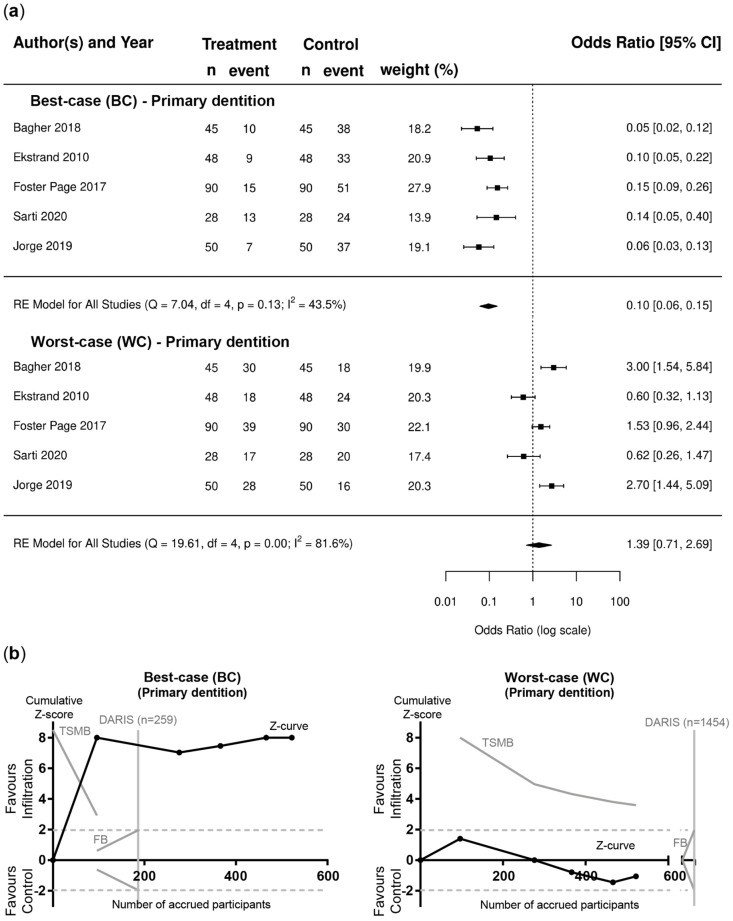
Primary dentition. (**a**) Forest plots of pairwise meta-analysis for BC and WC scenarios. Risk of failure, i.e., lesion progression, is indicated for lesions in primary teeth treated with resin infiltration and non-invasive measures compared to non-invasive measures alone. The odds ratio (OR) and 95% confidence intervals (CI) are shown. Heterogeneity is indicated by I^2^ and Q statistics. Diamonds indicate pooled effect estimates. The number of lesions was adjusted using the design effect as described. (**b**) Trial sequential analysis (TSA) of trials evaluating lesions in primary teeth [63,64,65,66,67] for BC and WC scenarios to assess the robustness of evidence. The cumulative Z-score (black curve), i.e., the accumulated level of significance, was plotted against the number of participants accrued so far, which was compared with the heterogeneity-adjusted required information size (DARIS), the conventional border (Z = 1.96), the trial sequential monitoring boundary (TSMB), and the futility border (FB) (grey oblique). For the BC scenarios, the Z-curve crossed the conventional border and TSMB before reaching DARIS, whereas for the WC scenario, the Z-curve failed to cross the conventional border, TSMB, and DARIS.

Evaluating the BC and WC scenarios, pairwise meta-analysis and TSA indicated the clear superiority of the resin infiltration group in the BC analysis for lesions in permanent teeth (OR [95%CI]; 0.12 [0.09–0.17]) (Figure 4a) and primary teeth (OR [95%CI]; 0.10 [0.06–0.15]) (Figure 5a). In contrast, for the WC scenario, no significant differences could be found for lesions in permanent teeth (OR [95%CI]; 1.17 [0.56–2.45]) (Figure 4a) and primary teeth (OR [95%CI]; 1.39 [0.71–2.69]) (Figure 5a). TSA confirmed that the required information size was reached in the BC scenario, whereas the Z-curves in the WC scenario failed to cross the conventional border, TSMB and DARIS (Figure 4b and Figure 5b). For the BC analysis, heterogeneity was not found in studies involving permanent dentition (I^2^ = 0.3%; Figure 4a), whereas moderate heterogeneity was observed in primary dentition studies (I^2^ = 43.5%; Figure 5a). However, substantial heterogeneity was found for WC scenario (I^2^ = 85.3% and 81.6%%; Figure 4a and Figure 5a).

### 3.5. GRADE Assessment

Based on this meta-analysis, resin infiltration would avoid the progression of 204–281 fewer lesions in permanent teeth and 230–372 fewer lesions in primary teeth per 1000 treated lesions in a PP scenario, whereas according to the ITT analysis, 125–256 fewer lesions in permanent teeth and 129–296 fewer lesions in primary teeth per 1000 treated lesions are expected to progress. In the BC scenario, 351–412 fewer lesions in permanent teeth and 441–578 fewer lesions in primary teeth per 1000 treated lesions can be expected to progress, whereas in contrast, 211 more to 105 fewer lesions in permanent teeth and 241 more to 80 fewer lesions in primary teeth per 1000 treated lesions would progress in the WC scenario (Table 1).

Based on judged overall risk of bias, imprecision, and the magnitude of the effect estimates, the certainty of the evidence was graded as high for both lesions in primary and permanent teeth in the PP, ITT, and BC scenarios. For WC scenarios, the evidence was very low for lesions in both dentitions. In all of the scenarios, the risk of bias was assessed as ‘serious’ as all of the included studies presented an overall high or unclear risk of bias, whereas a downgrading of the certainty of the evidence due to inconsistency and imprecision was only necessary for WC scenarios due to substantial heterogeneity between the studies as well as small effect estimates and information sizes (Table 1).

## 4. Discussion

The identified, most recent body of evidence on the efficacy of resin infiltration is consistent with that identified in previous meta-analyses [17,18,19,21,23]. Resin infiltration in conjunction with non-invasive treatments is an effective therapeutic approach to manage non-cavitated proximal caries lesions in both primary and permanent teeth. The accrued evidence was found to be robust and reliable. With the inclusion of newer follow-up reports, the long-term efficacy of the treatment was confirmed as lesion progression was followed over 36 months for most trials assessing lesions in permanent teeth, with one study even following up over 7 years [59]. Studies involving primary teeth were limited in their follow-up by exfoliation, typically concluding after 24 months.

Based on GRADE, the certainty of the evidence was evaluated as high in the PP and ITT analyses, despite downgrading due to a high or unclear risk of bias in all of the studies. No downgrading was performed for inconsistency and imprecision, as there was no statistical heterogeneity between the studies and the TSA analyses, indicating that sufficient evidence has been accrued. Furthermore, no downgrading was performed on indirectness. While the method for outcome assessment differed between studies, i.e., DSR, pairwise reading, or lesion staging, significant bias was assumed as unlikely [53,54]. Publication bias could not be detected in the Funnel plot analysis. However, fewer than 10 studies were included per analysis, thus limiting the information value of asymmetry testing. [69]

A limitation of previous meta-analyses is the lack of a sensitivity analysis, i.e., an in-depth evaluation of the impact of missing data (attrition). It has been argued that attrition may not affect the overall risk of bias considering that studies on resin infiltration of proximal caries use a split-mouth design [16,19]. To further validate the impact of the missing data, we performed a meta-analysis with an ITT scenario, assuming that all of the missing participants experienced progression of treated and control lesions. Due to the split-mouth design of the included studies, the number of failed lesions increases evenly in both groups, which in turn increases the risk of failure in both groups accordingly. As a consequence, the relative effect size shrinks significantly (permanent teeth OR; PP: 0.24 vs. ITT: 0.44; primary teeth OR; PP: 0.28 vs. ITT: 0.41). However, resin infiltration in combination with non-invasive treatments was still highly favored compared to non-invasive treatments alone in an ITT scenario despite the high percentage of drop-outs, indicating a large treatment effect and the robustness of the results. Despite this somewhat extreme scenario (considering the high drop-out rate), the conclusions were found to be based on quantitatively (indicated by TSA) and qualitatively (high certainty indicated by GRADE) sufficient data.

In addition to PP and ITT, BC/WC scenarios were computed as part of the sensitivity analysis. While these are not the most ideal strategies for dealing with missing values, they can offer insights on data reliability and robustness as they are the most extreme and conservative scenarios related to uncertainty caused by missing data [35,36,70]. Considering the high percentage of drop-outs in the included studies together with the already favorable outcome for resin infiltration in PP and ITT analysis, it is of no surprise that resin infiltration was even more favorable in the BC analysis. In contrast, the WC analysis indicated no differences between resin infiltration and the control for lesions in permanent (OR [95%CI]; 1.17 [0.56–2.45]) and primary teeth (OR [95%CI]; 1.39 [0.71–2.69]). However, TSA and GRADE suggested that the result was neither based on qualitatively or quantitatively sufficient data. Albeit being too extreme for meaningful analysis, the WC scenario still indicated that the worst that can be expected in the most extreme circumstance is a similar efficacy between resin infiltration in combination with non-invasive methods and non-invasive methods alone, while superior performance of resin infiltration can be expected in all other scenarios.

This study comes with a number of strengths. First, to our knowledge, this is the first meta-analysis on resin infiltration where sensitivity analyses were performed to assess the robustness and certainty of the results. Second, this study was conducted systematically, incorporating new follow-up reports of previous trials, most notably of the trial with the largest cohort [57], thus providing further insights on long-term performance. Third, all of the included studies were RCTs and used a split-mouth design, and there was no statistically significant heterogeneity among the enrolled studies, which promotes certainty in the results. Fourth, analyses in this review followed a robust and detailed methodology taking the clustering and correlation of the split-mouth data into consideration [23], thus providing more reliable results for pooled estimates and confidence intervals. Fifth, with this being an updated meta-analysis, TSA were applied to account for repeated testing as well as to assess whether conclusions from different scenarios are based on qualitatively sufficient data. We hereby confirmed a previous conclusion, i.e., that the accrued evidence on resin infiltration is convincing and that additional studies to assess general efficacy are not needed [23]. Moreover, we delineated that this holds true for the primary and permanent dentition as well as the PP and ITT scenarios. Sixth, this review specifically focused on the effectiveness of resin infiltration in primary and permanent teeth, thus enabling a robust assessment of the treatment effectiveness. In addition, while only one practice-based trial was included [57], conclusions can still be generalized to some extent to routine settings as all of the studies used the commercial product “Icon” (DMG, Hamburg) or its pre-market release variant with the standardized application.

This study also comes with some limitations. First, we did not evaluate the efficacy of resin infiltration based on different caries risk levels as there is insufficient data to conclude with certainty [19]. Subgroup analysis would have been underpowered, likely generating false or uncertain conclusions, illustrating the need for further studies to generate sufficient evidence. Second, all of the included studies presented an unclear or high risk of bias, with allocation concealment and operator blinding being the two main sources. While all of the studies on permanent teeth used a placebo treatment for the control, likely achieving participant blinding if not operator blinding as well, this was not carried out with primary teeth to avoid unnecessary stress and treatment time for the children. However, it has been argued that children are less influenced in their daily routine by not being blinded [67]. Third, this review included studies with various radiographic outcome assessments (i.e., DSR, pairwise reading, and lesion staging). While there are differences in sensitivity between the assessment modalities, significant bias was assumed to be unlikely [53,54]. Nonetheless, a standardized detection method, preferably DSR as the most sensitive, would have been more optimal. Fourth, this review was prone to industry-related bias as two of the authors are affiliated with the company that is marketing the commercial resin infiltrate Icon and one of the inventors of Icon is an author as well. However, to minimize bias, this review was registered and conducted systematically, including robust and detailed methodology as well as transparent reporting. Furthermore, the results and conclusions are well in line with previous meta-analyses [17,18,19,21,23].

## 5. Conclusions

In summary, resin infiltration in combination with non-invasive treatments is more efficacious than non-invasive treatments alone in treating proximal caries lesions in both primary and permanent teeth. The accrued evidence is robust and convincing, and there is high certainty in the results. Future studies should focus on aspects such as caries risk, cost-effectiveness, or setting-specific differences in efficacy or applicability.

## Figures and Tables

**Table 1:** Summary of findings table according to GRADE. Evidence supporting resin infiltration and non-invasive measures versus non-invasive measures alone in preventing lesion progression in permanent and primary teeth has been assessed. The risk of lesion progression in the test group and the control group, the relative effects based on the number of studies, and assessed lesions are given.

Resin Infiltration Compared to Control for Proximal Caries Lesions.						
**Patient or Population:** **Intervention:** **Comparison:**		Non-Cavitated Proximal Caries Lesions in Primary or Permanent Teeth Resin Infiltration + Non-Invasive Measures Non-Invasive Measures				
**Outcomes**	Anticipated absolute effects * (95% CI)			**Relative effect** **(95% CI)**	**No. of** **lesions** **(studies)**	**Certainty of** **the evidence** **(GRADE)**
	Risk with **Non-invasive**	Risk with **Resin Infiltration + non-invasive**	Risk difference with **Resin Infiltration** ** + non-invasive**			
**Per-protocol (PP) scenario**						
Permanent teeth	372 per 1.000	125 per 1.000 (92 to 168)	**248 fewer per 1.000** (281 fewer to 204 fewer)	**OR 0.24** (0.17 to 0.34)	564 (6 RCTs)	⨁⨁⨁⨁ High ^a,b^
Primary teeth	581 per 1.000	279 per 1.000 (208 to 351)	**301 fewer per 1.000** (372 fewer to 230 fewer)	**OR 0.28** (0.19 to 0.39)	370 (5 RCTs)	⨁⨁⨁⨁ High ^a,b^
**Intention-to-treat (ITT) scenario**						
Permanent teeth	493 per 1.000	300 per 1.000 (237 to 368)	**193 fewer per 1.000** (256 fewer to 125 fewer)	**OR 0.44** (0.32 to 0.60)	702 (6 RCTs)	⨁⨁⨁⨁ High ^a,b^
Primary teeth	701 per 1.000	490 per 1.000 (405 to 572)	**211 fewer per 1.000** (296 fewer to 129 fewer)	**OR 0.41** (0.29 to 0.57)	522 (5 RCTs)	⨁⨁⨁⨁ High ^a,b^
**Best-case (BC) scenario**						
Permanent teeth	493 per 1.000	104 per 1.000 (80 to 142)	**388 fewer per 1.000** (412 fewer to 351 fewer)	**OR 0.12** (0.09 to 0.17)	702 (6 RCTs)	⨁⨁⨁⨁ High ^a,c^
Primary teeth	701 per 1.000	190 per 1.000 (123 to 260)	**511 fewer per 1.000** (578 fewer to 441 fewer)	**OR 0.10** (0.06 to 0.15)	522 (5 RCTs)	⨁⨁⨁⨁ High ^a,c^
**Worst-case (WC) scenario**						
Permanent teeth	296 per 1.000	330 per 1.000 (191 to 508)	**34 more per 1.000** (105 fewer to 211 more)	**OR 1.17** (0.56 to 2.45)	702 (6 RCTs)	⨁◯◯◯ Very low ^a,d,e,f^
Primary teeth	414 per 1.000	495 per 1.000 (334 to 655)	**81 more per 1.000** (80 fewer to 241 more)	**OR 1.39** (0.71 to 2.69)	522 (5 RCTs)	⨁◯◯◯ Very low ^a,d,e,g^
* The risk in the intervention group (and its 95% confidence interval) is based on the assumed risk in the comparison group and the relative effect of the intervention (and its 95% CI). **CI:** confidence interval; **OR:** odds ratio						
**Grade Working Group grades of evidence** **High certainty:** we are very confident that the true effect lies close to that of the estimate of the effect. **Moderate certainty:** we are moderately confident in the effect estimate: the true effect is likely to be close to the estimate of the effect, but there is a possibility that it is substantially different.						
^a^ High or unclear risk of bias in all studies (Risk of bias: serious) ^b^ Large effect (OR < 0.5) ^c^ Very large effect (OR < 0.2) ^d^ No large effect (0.5 < OR < 2.0) ^e^ Substantial heterogeneity (I^2^ > 80%) ^f^ CI crosses the clinical decision threshold & DARIS not reached (see Figure 4b and Figure 5b) (Imprecision: very serious) ^g^ DARIS not reached (see Figure 4b and Figure 5b) (Imprecision: serious)						

## Data Availability

Not applicable.

## Supplementary Materials

The following supporting information can be downloaded at: https://www.mdpi.com/article/10.3390/jcm12020727/s1. Table S1: Characteristics of included studies; Table S2: Risk of bias of included studies; Figure S1: Funnel plot analysis.
