# Supplementary material for: Resin Infiltration of Non-Cavitated Proximal Caries Lesions in Primary and Permanent Teeth: A Systematic Review and Scenario Analysis of Randomized Controlled Trials
Source: J Clin Med. 2023 Jan 16;12(2):727. doi: 10.3390/jcm12020727 (PMC9864315; doi:10.3390/jcm12020727)
Supplement: Supplementary file 1 [file jcm-12-00727-s001.zip › jcm-2134041-supplementary.pdf]

Supplementary Information

Table S1. Characteristics of included studies

Study	Intervention	Control	Concomitant treatment (both groups)	Funding	Country	Setting	Follow up (months)	Patients treated	Patients examined	Lesions treated	Mean age	Caries risk	Assessment	Intervention	Control
														progressed/ assessed	
Permanent Dentition															
[a,c]Meyer-Lückel 2021 (Meyer-Lückel 2016) [1, 2]	Resin infiltration (Icon, DMG)	Sham infiltration	Oral hygiene and dietary instruction + fluoride varnish	DMG	Germany	Dental practices	36	75	64	390	23	Moderate 29%<br>High 71%	Pairwise reading	23/165	64/165
Arslan 2020 [3]	Resin infiltration (Icon, DMG)	-	Oral hygiene and dietary instruction	Public	Turkey	Dental school	12	56	41	120[d]	21	Low (12%)<br>Moderate (54%)<br>High (34%)	Digital subtraction radiography	1/45	9/45
[a,b]Paris 2020 (Meyer-Lückel 2012, Paris 2010) [4-6]	Resin infiltration (Icon pre-product, DMG)	Sham infiltration	Oral hygiene and dietary instruction	DMG	Germany	Dental school	84	22	16	58	25	Low (32%)<br>Moderate (36%)<br>Increased (23%)<br>High (9%)	Digital subtraction radiography	2/22	10/22
Peters 2019 (Peters 2018) [7, 8]	Resin infiltration (Icon, DMG)	Sham infiltration	Oral hygiene and dietary instruction + fluoride varnish	Public and DMG	USA	Military academy dental clinic	36	42	29	84	20	High (100%)	Pairwise reading	3/29	7/29
Arthur 2018 [9]	Resin infiltration (Icon, DMG)	Sham infiltration	Oral hygiene and dietary instruction + topical fluoride	Public	Brazil	Dental school	36	36	27	72	26	n/a	Pairwise reading	2/27	5/27
[b]Martignon 2012 [10]	Resin infiltration (Icon pre-product, DMG)	Sham infiltration	Flossing advice	DMG	Columbia	Dental school	36	39	37	78	21	Low (46%)<br>Moderate (28%)<br>High (21%)<br>Very high (5%)	Digital subtraction radiography	12/37	26/37
Primary Dentition															
Sarti 2020 [11]	Resin infiltration (Icon, DMG)	-	Oral hygiene and dietary instruction + topical fluoride	n/a	Brazil	Dental school	24	28	24	56	6	Moderate (54%)<br>High (46%)	Lesion staging	13/24	20/24
Jorge 2019 (Ammari 2017) [12, 13]	Resin infiltration (Icon, DMG)	-	Oral hygiene instruction	Public and DMG	Brazil	Dental school	24	50	29	100	6	Low (6%)<br>Moderate (46%)<br>High (48%)	Pairwise reading	7/29	16/29

Bagher 2018 [14]	Resin infiltration (Icon, DMG)	-	Oral hygiene and dietary instruction + fluoride varnish	n/a	USA	Dental school	24	45	25	90	7	Low (31%) Moderate (8%) High (51%)	Pairwise reading	10/25	18/25
Foster-Page 2017 [15]	Resin infiltration (Icon, DMG)	-	Preventive care + fluoride varnish	Public and DMG	New Zealand	Communi ty clinics	24	90	69	180	8	Low (58%) Moderate (42%)	Pairwise reading	15/66	30/69
[b]Ekstrand 2010 [16]	Resin infiltration (Icon pre-product, DMG)	-	Oral hygiene instruction + fluoride varnish	DMG	Green-land	Public health clinic	12	48	39	96	7	Low (12%) Moderate (21%) High (67%)	Lesion staging	9/39	24/39

[a] Studies conducted by the inventors.

[b] Studies conducted before market release.

[c] The latest follow up time point for the whole study group was at 36 month, whereas only subgroup (high caries risk patients) were followed up for 48 months. Consequently, the 36 month follow up was defined at the latest time point to be included in the meta-analysis.

[d] The number of treated lesions (of the 56 patients at baseline) was not reported and the authors of this study could not be reached for clarification. In order to enable inclusion of this study into the meta-analysis we thus assumed that one lesion pair per patient (for the 15 drop outs) had been included. This assumption is supported by the fact that of 41 patients that were followed up, 38 patients had 1 lesion pair included in this study and only 2 patients 2 lesion pairs and 1 patient 3 lesion pairs.

n/a … not available

Table S2. Risk of bias of included studies, followed guidelines outline in the Cochrane Handbook for Systematic Reviews of Interventions [17]

Study	Sequence generation	Allocation concealment	Blinding of personnel and participants	Blinding of outcome assessment	[e]Incomplete outcome data	Selective reporting	Other bias
Permanent Dentition							
[a]Meyer-Lückel 2021 (Meyer-Lückel 2016) [1, 2]	Low (computer generated permuted blocks)	Low (sealed envelopes)	Unclear (participants blinded, sham treatment)	Low (examiner not blinded, but likely unaware of treatment group)	Low (15% dropped out, reason for drop-out reported)	Unclear (no protocol available)	Low
Arslan 2020 [3]	Low (Coin toss (if 2 lesions present) / Lottery method (if >2 lesions present))	Unclear (not stated who performed randomization)	High (personnel and participants not blinded)	Low (blinded)	Unclear (27% dropped out, reasons for drop-out not reported)	Low	Unclear[d] (number of treated lesions not reported)
[a,b]Paris 2020 (Meyer-Lückel 2012, Paris 2010) [4-6]	Low (computer generated permuted blocks)	Low (sealed envelopes)	Unclear (participants blinded, sham treatment)	Low (blinded external examiner)	Low (24% dropped out, reason for drop-out reported)	Unclear (no protocol available)	Low
Peters 2019 (Peters 2018) [7, 8]	Low (computer derived randomization)	Low (concealed until treatment)	Unclear (participants blinded, sham treatment)	Low (blinded)	Unclear (31% dropped out, reasons for drop-out not reported)	Unclear (assessment used for primary endpoint differs from protocol)	Low
Arthur 2018 [9]	Low (computer generated random numbers table)	Unclear (allocation concealment not reported)	Unclear (participants blinded, sham treatment)	Low (blinded)	Unclear (25% dropped out, reasons for drop-out not reported)	Unclear (no protocol available)	Low
[b]Martignon 2012 [10]	Low (computer generated permuted blocks)	Unclear (allocation concealment not reported)	Unclear (participants blinded, sham treatment)	Low (independent and blinded)	Low (5% dropped out)	Low	Low
Primary Dentition							
Sarti 2020 [11]	Low (computer generated permuted blocks)	Unclear (allocation concealment not reported)	High (personnel and participants not blinded)	Low (independent and blinded)	Low (14% dropped out, reasons for drop-out not reported)	Unclear (no protocol available)	Low
Jorge 2019 (Ammari 2017) [12, 13]	Low (Coin toss (to select group) / Lottery method (to select lesions if >2 lesions were present))	Unclear (allocation concealment not reported)	High (personnel and participants not blinded)	Low (blinded)	Unclear (42% dropped out, reasons for drop-out reported)	Low	Low

Bagher 2018 [14]	Low (computer generated scheme)	Low (concealed until treatment)	High (personnel and participants not blinded)	Low (blinded)	Unclear (44% dropped out, reasons for drop-out reported)	Unclear (no protocol available)	Low
Foster-Page 2017 [15]	Low (computer generated permuted blocks)	Low (sealed envelopes)	High (not stated, but personnel and participants likely not blinded)	Low (blinded)	Low (23% dropped out, reasons for drop-out reported)	Low	Low
[b]Ekstrand 2010 [16]	Low (random number table)	Low (pre-prepared list)	Unclear (participants blinded, personnel not blinded)	Low (blinded)	Low (19% dropped out, reasons for drop-out reported)	Unclear (no protocol available; 6 month data not reported)	Unclear (unbalanced severity to the expense of the test group)

[a] Studies conducted by the inventors.
[b] Studies conducted before market release.
[c] The 48 month follow up was only reported for a subgroup (high caries risk). Data included in the meta-analysis were the 36 month follow up. See Table S1 for further information.
[d] The number of lesions treated at baseline was not reported and the authors could not be reached for clarification. See Table S1 for further information.
[e] Low risk of bias was assumed if drop-out was below 25%. Unclear risk of bias was assumed if drop-out rate was above 25% and no information on reasons were reported, but attrition was balanced. High risk of bias was assumed if drop-out was above 25% and groups were unbalanced. [18]

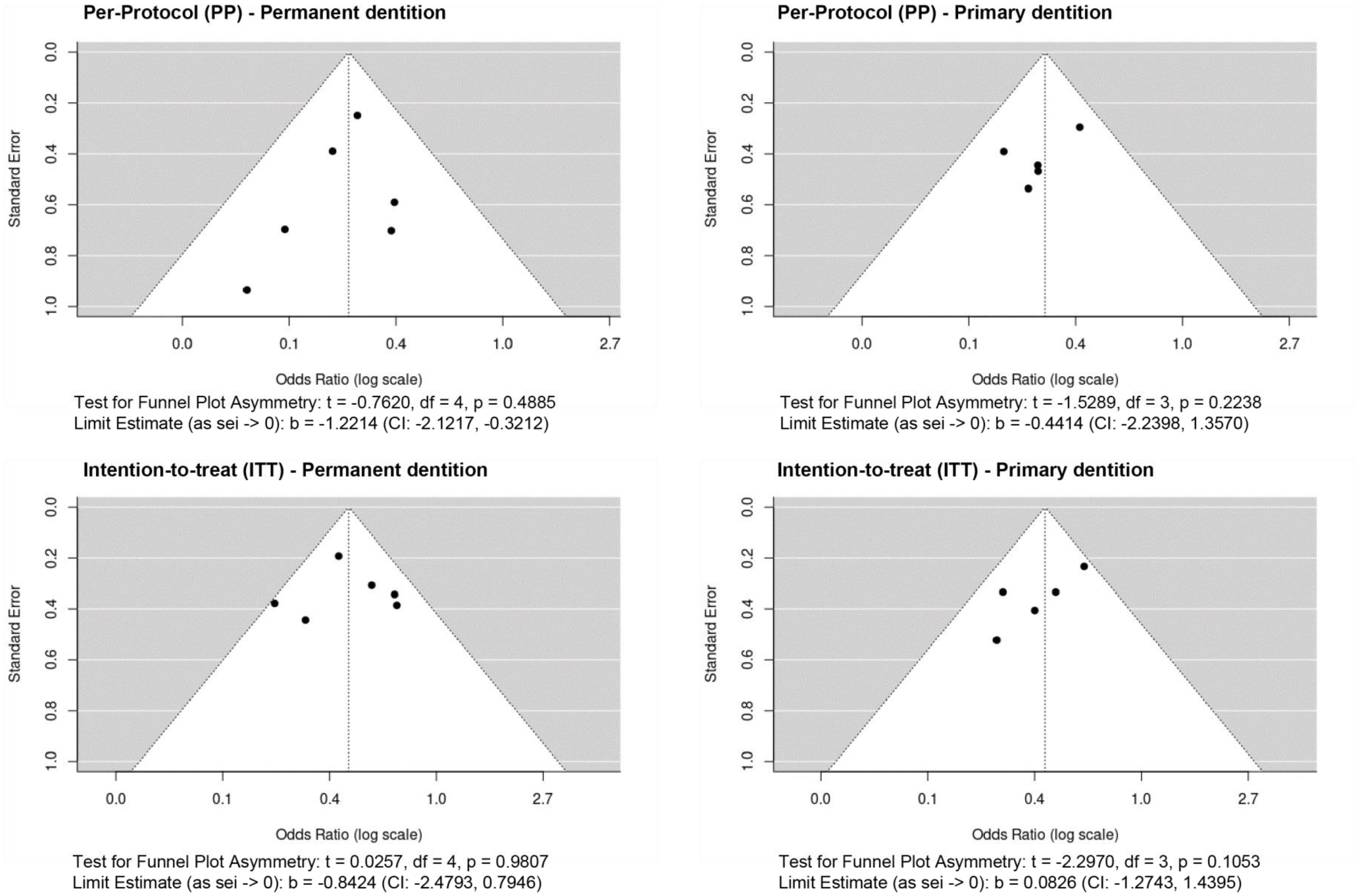

Figure S1: Funnel plot analysis for PP and ITT scenarios for the permanent (left) and primary (right) dentition.

References

[1] H. Meyer-Lueckel, A. Wardius, J. Krois, K. Bitter, C. Moser, S. Paris, R.J. Wierichs, Proximal caries infiltration - Pragmatic RCT with 4 years of follow-up, Journal of dentistry 111 (2021) 103733.

[2] H. Meyer-Lueckel, A. Balbach, C. Schikowsky, K. Bitter, S. Paris, Pragmatic RCT on the Efficacy of Proximal Caries Infiltration, J Dent Res 95(8) (2016) 531-6.

[3] S. Arslan, M.H. Kaplan, The Effect of Resin Infiltration on the Progression of Proximal Caries Lesions: A Randomized Clinical Trial, Medical principles and practice : international journal of the Kuwait University, Health Science Centre 29(3) (2020) 238-243.

[4] S. Paris, K. Bitter, J. Krois, H. Meyer-Lueckel, Seven-year-efficacy of proximal caries infiltration - Randomized clinical trial, Journal of dentistry 93 (2020).

[5] H. Meyer-Lueckel, K. Bitter, S. Paris, Randomized controlled clinical trial on proximal caries infiltration: three-year follow-up, Caries research 46(6) (2012) 544-8.

[6] S. Paris, W. Hopfenmuller, H. Meyer-Lueckel, Resin infiltration of caries lesions: an efficacy randomized trial, J Dent Res 89(8) (2010) 823-6.

[7] M.C. Peters, A.R. Hopkins, Jr., L. Zhu, Q. Yu, Efficacy of Proximal Resin Infiltration on Caries Inhibition: Results from a 3-Year Randomized Controlled Clinical Trial, J Dent Res 98(13) (2019) 1497-1502.

[8] M.C. Peters, A.R. Hopkins, Jr., Q. Yu, Resin infiltration: An effective adjunct strategy for managing high caries risk-A within-person randomized controlled clinical trial, Journal of dentistry 79 (2018) 24-30.

[9] R.A. Arthur, J.E. Zenkner, J.C. d'Ornellas Pereira Junior, R.T. Correia, L.S. Alves, M. Maltz, Proximal carious lesions infiltration-a 3-year follow-up study of a randomized controlled clinical trial, Clinical oral investigations 22(1) (2018) 469-474.

[10] S. Martignon, K.R. Ekstrand, J. Gomez, J.S. Lara, A. Cortes, Infiltrating/sealing proximal caries lesions: a 3-year randomized clinical trial, J Dent Res 91(3) (2012) 288-92.

[11] C.S. Sarti, M.B. Vizzotto, L.V. Filgueiras, C.C. Bonifácio, J.A. Rodrigues, Two-Year Split-Mouth Randomized Controlled Clinical Trial on the Progression of Proximal Carious Lesions on Primary Molars After Resin Infiltration, Pediatric dentistry 42(2) (2020) 110-115.

[12] R.C. Jorge, M.M. Ammari, V.M. Soviero, I.P.R. Souza, Randomized controlled clinical trial of resin infiltration in primary molars: 2 years follow-up, Journal of dentistry 90 (2019) 103184.

[13] M.M. Ammari, R.C. Jorge, I.P.R. Souza, V.M. Soviero, Efficacy of resin infiltration of proximal caries in primary molars: 1-year follow-up of a split-mouth randomized controlled clinical trial, Clinical oral investigations 22(3) (2017) 1355-1362.

[14] S.M. Bagher, F.M. Hegazi, M. Finkelman, A. Ramesh, N. Gowharji, G. Swee, O. Felemban, C.Y. Loo, Radiographic Effectiveness of Resin Infiltration in Arresting Incipient Proximal Enamel Lesions in Primary Molars, Pediatric dentistry 40(3) (2018) 195-200.

[15] L.A. Foster Page, A. Beckett, R. Ahmadi, D.R. Schwass, S. Leon de la Barra, S.M. Moffat, A. Meldrum, W.M. Thomson, Resin Infiltration of Caries in Primary Molars in a Community Setting: 24-Month Randomized Controlled Trial Findings, JDR Clin Trans Res 2(3) (2017) 287-294.

[16] K.R. Ekstrand, A. Bakhshandeh, S. Martignon, Treatment of proximal superficial caries lesions on primary molar teeth with resin infiltration and fluoride varnish versus fluoride varnish only: efficacy after 1 year, Caries research 44(1) (2010) 41-6.

[17] J.P.T. Higgins, D.G. Altman, J.A.C. Sterne, Chapter 8: Assessing risk of bias in included studies, Cochrane Handbook for Systematic Reviews of Interventions version 5.2.0 (updated June 2017), Cochrane 2017.

[18] M. Dorri, S.M. Dunne, T. Walsh, F. Schwendicke, Micro-invasive interventions for managing proximal dental decay in primary and permanent teeth, The Cochrane database of systematic reviews 2015(11) (2015) Cd010431.
